# Is syncope a predictor of mortality in acute pulmonary embolism?

**DOI:** 10.25122/jml-2018-0063

**Published:** 2019

**Authors:** Rodica Lucia Ploesteanu, Alexandru Cristian Nechita, Silvia Andrucovici, Caterina Delcea, Elena Mihaela Mihu, Diana Gae, Sorin Costel Stamate

**Affiliations:** 1. Sf. Pantelimon Emergency Hospital, Bucharest, Romania; 2. Carol Davila University of Medicine and Pharmacy, Faculty of Medicine, Bucharest, Romania

**Keywords:** Acute pulmonary embolism, syncope, hemodynamic instability, thrombotic load, echocardiography

## Abstract

Whether syncope as a presenting symptom independently classifies acute pulmonary embolism (APE) into a high mortality risk group remains a matter of controversy.

We retrospectively included all consecutive patients admitted to our clinic with APE from January 2014 to December 2016.

Our sample consisted of 76 patients with a mean age of 69 ±13.6 years, 64.5% female. 14.3% presented with syncope at admission. In-hospital mortality was 20.8%. Patients with syncope were more likely to require inotropic support (OR = 5.2, 95 % 1.17-23.70, p=0.03) due to the association of cardiogenic shock (OR= 15.95% CI 3.02-74.32, p < 0.001) and systolic blood pressure below 90 mmHg (OR=5.52, 95% CI 1.24-24.47, p=0.03). Patients with syncope had a higher PESI score (150.9 ± 51.1 vs 99.9 ± 30.1, p < 0.001) and a greater in-hospital mortality (OR= 4.5, 95% CI 1.14-17.62, p=0.03). However, multivariate logistic regression equations did not identify syncope as an independent predictor of mortality.

In our sample, syncope did not independently reclassify the patient in a higher mortality group, but due to the association with hemodynamic instability, which remains the primary tool in therapeutic decision-making.

## Introduction

Acute pulmonary embolism (APE) is a condition with a high fatality rate mainly due to the variability of onset symptoms that can delay diagnosis, ranking third as one of the most frequently occurring cardiovascular diseases with an overall annual incidence of 100–200 per 100.000 inhabitants [1].

Syncope represents the onset symptom in 9 to 35% of patients [2–6]. Whether this symptom will better help the attending physician to classify the patient into a higher risk group or not, remains a matter of controversy as currently available data does not show a significant correlation with mortality [7–9].

APE mortality rate varies between 1% and 60%, depending on the clinical presentation and the severity of right ventricular dysfunction [10,11]. It is therefore essential to promptly and accurately stratify the patient’s risk in order to optimize therapy immediately after admission [12,13]. The risk stratification strategy is now primarily based on clinical features and secondarily on right ventricular myocardial dysfunction and/or myocardial injury [10].

Life-threatening causes of syncope are especially of cardiovascular origin (including APE) [14]. The classic symptoms of APE include chest pain, dyspnea, and hemoptysis. APE was identified in 2.2% up to 35% of patients with syncope [3–5,15–18]. Sometimes the critical state and the quality of the patient’s medical history taking does not enable the physician to distinguish true syncopal events from other states of transient loss of consciousness so it may be reasonable to doubt whether all syncopal cases reported in the setting of APE meet the diagnostic criteria.

Hemodynamic alterations represent one of the most prevalent mechanisms of syncope in the setting of APE. Acute right ventricular (RV) failure causes a drop in stroke volume and cardiac output followed by secondary systemic hypotension. This process is enhanced by myocardial ischemia and hypodiastolic filling of the left ventricle (LV) due to excessive RV overload. In this condition, additional excessive tachy- or bradyarrhythmias can also induce syncope. The spontaneous recovery from syncope with reasonable cardiac and cerebral perfusion presumes those conditions are very often transitory. Syncope can appear at the onset of significant pulmonary artery obstruction while afterward, through adaptive mechanisms, cardiac function can become stable. As for systemic hypotension, only 13% of PE patients with syncope enrolled in the ICOPER registry were hypotensive [17].

Metabolic changes, such as hypocapnia and hypoxemia, could also explain the appearance of syncope in APE cases. Reflex mechanisms were also suggested as a possible cause of syncope in APE.

It is important to highlight that more than a single mechanism may be implicated in a single patient.

There are conflicting data concerning syncope and the prognosis of APE. In earlier studies, the presence of a syncopal episode at hospital admission was reported to be associated with a high risk of mortality [4,19] in patients with APE. However, this was not confirmed in recent studies [6,7,20]. It is essential to determine whether syncope is associated with poor prognosis as it allows prompt risk stratification and appropriate treatment in this subset of patients. This study was performed in order to determine the clinical and prognostic characteristics of patients with APE and syncope.

## Materials and Methods

We retrospectively analyzed the medical records of all patients diagnosed with APE admitted to the Cardiology Department of the “Sf. Pantelimon” Emergency Hospital in Bucharest, Romania.

The study protocol was reviewed and approved by the Ethics Committee number 49/2018 of the “Sf. Pantelimon”Emergency Hospital. On admission, the patients included in this study signed a consent form agreeing that their medical records could be used anonymously for research purposes.

### Study Population

We included all consecutive adult patients with APE hospitalized in our clinic from January 1st, 2014 to december 31st, 2016, diagnosed with the use of CT pulmonary angiogram (CT) according to the guidelines of the European Society of Cardiology (ESC) [12].

Patients without imaging confirmation of APE diagnosis were excluded.

## Definitions

Syncope, according to the 2009 and 2018 ESC guidelines, is a transient loss of onsciousness due to brief global cerebral hypoperfusion [14]. It is characterized by rapid onset, short duration, and complete spontaneous recovery [14].

We used troponin I levels to diagnose cardiac injury (increased above the 99th percentile of normal). Right ventricular dysfunction (RVD) was defined as the presence of at least one of the following signs: RV enlargement (expressed as RV diameter/LV diameter > 0.9 in the four-chamber view in transthoracic echocardiography or CT) and/or decreased tricuspid annular plane systolic excursion (TAPSE) below 16 mm [21].

Systolic blood pressure below 90 mmHg at admission or a pressure drop of more than 40 mmHg for longer than 15 minutes, were the parameters used to classify the patients as high-risk according to the definition from the ESC guidelines [12]. The Shock Index (SI) was calculated as heart rate divided by systolic blood pressure.

Thrombus localization on computed tomography of the chest was classified as peripheral (isolated lobar, segmental, and/or subsegmental arteries) or central [22].

### Clinical and paraclinical parameters

Clinical, biological and imaging data at the time of admission as well as in-hospital mortality status were obtained from hospital records. Information regarding medium-term survival was gathered from telephone interviews and the national healthcare database in January 2017. Regarding survival status, the accessible data referred to all-cause mortality.

The clinical parameters collected included age, sex, presenting symptoms (dyspnea, chest pain, syncope), blood pressure, heart rate, respiratory rate, oxyhemoglobin saturation, cardiogenic shock or cardiopulmonary resuscitation.

Laboratory panel used for analysis comprised of arterial blood gas analysis, lactate levels (mg/dl), troponin I, serum creatinine, serum sodium, serum potassium, hemoglobin levels, leucocyte and platelet counts.

From the electrocardiogram, we recorded the presence of the S_1_Q_3_T_3_ pattern or ST-T changes, the duration of the QRS complex, and the corrected QT interval. The main echocardiographic parameters used for analysis included right heart chambers’ sizes, right ventricle to right atrium gradient and TAPSE. Information about the localization of the thrombus on the chest CT scan was recorded. A Doppler ultrasound was used to diagnose and localize deep vein thrombosis. The therapy provided to our patients was documented, including the use of fibrinolytic agents, inotropes and anticoagulants.

### Statistical analysis

Statistical analysis was performed using IBM SPSS Statistics 20 and MedCalc Statistical Software.

We used numeric and categorical parameters. Bartlett’s test for homogeneity was used to determine if variances are equal between compared variables. Homogeneous numerical variables were expressed as means ± standard deviations and comparisons were made employing the ANOVA parametric test. Inhomogeneous numerical variables were expressed as median (25% percentile; 75% percentile) and compared using the Mann-Whitney Wilcoxon two-sample test.

Categorical parameters were expressed as percentages, and associations were assessed with the Chi-squared corrected test and expressed as odds ratios (OR) with 95% confidence intervals (95%CI).

To determine the use of numerical variables as predictors of outcome, we employed ROC curve analysis to calculate the integrals of the curves expressed as area under the curve (AUC) and 95%CI.

Multivariate logistic regression models, using the Enter method, were utilized to identify independent predictors of outcome and hazard ratios (HR) with 95% confidence intervals were computed for each variable included in the models.

All P-values were two-sided, and values less than 0.05 were considered statistically significant.

## Results

Our study group consisted of 76 patients with a mean age of 69 ± 13.6 years. 14.3% presented with syncope on admission to the hospital. The patients’ clinical characteristics are listed in Table 1.

**Table 1:** Patient characteristics

	All (n=76)	Without syncope (n=65)	With syncope (n=11)	p value
**General Characteristics**				
Age (years)	69 ± 13.6	68.6 ± 13.7	72.4 ± 13.4	0.40
Sex (male), n	27 (35%)	21 (32%)	6 (54%)	0.16
**Malignancy**, n	13 (17%)	10 (15%)	3 (27%)	0.32
**Clinical Parameters**				
Dyspnea, n	64 (83%)	58 (87%)	6 (54%)	< 0.01
Chest pain, n	20 (26%)	19 (30%)	1 (9%)	0.14
HR (bpm)	96.9 ± 23.3	96.2 ± 23.2	101.0 ± 25.1	0.53
SBP ≤ 90 mmHg, n	10 (13%)	6 (9%)	4 (36%)	0.01
Shock index	0.8 ± 0.3	0.7 ± 0.2	1.1 ± 0.5	< 0.01
**Laboratory Parameters**				
Troponin I value (ng/ml)	2.1 ± 8.7	1 ± 1.2	8.3 ± 21	0.92
Hemoglobin (g/dl)	13.5 ± 1.9	13.5 ± 2.1	13.2 ± 1.9	0.58
Creatinine (mg/dl)	1 ± 0.3	1 ± 0.3	1.1 ± 6.5	0.31
**Central localization of thrombus**, n	24 (51%)	22 (52%)	2 (40%)	0.46
**Right ventricular dysfunction**, n	19 (47%)	15 (44%)	4 (6%)	0.02
**Treatment**				
Thrombolysis, n	16 (21%)	10 (16%)	6 (54%)	< 0.01
Inotropic therapy, n	18 (24%)	12 (18%)	6 (55%)	< 0.01
**Outcomes**				
In-hospital mortality, n	16 (21%)	11 (17%)	5 (45%)	0.044
Medium-term mortality, n	11 (18%)	10 (19%)	1 (17%)	0.91

HR – heart rate, SBP – systolic blood pressure.

In-hospital mortality was 20.8% with a mean duration of hospitalization of 11 days ± 8.5. Of the 61 patients surviving the initial thromboembolic event, medium-term mortality was 18.64%.

On admission, patients with syncope associated less frequently dyspnea than the control group (p = 0.008). However, they expressed increased respiratory effort by a higher respiratory rate (43 respirations per minute ± 2.1 versus 24.5 respirations per minute ± 8.0, p<0.001).

In the ROC curve analysis, the syncope was associated with lower SBP and DBP, higher SI, but not with increased heart rate or decreased oxyhemoglobin saturation (Table 2). Of the biological parameters, lactate levels correlated with the presence of syncope.

**Table 2:** ROC curve analysis for syncope correlations

	AUC (95%CI)	p value
**Clinical examination**		
SBP (mmHg)	0.835 (0.733–0.910)	< 0.01
DBP (mmHg)	0.713 (0.594–0.813)	0.01
HR (bpm)	0.543 (0.426–0.657)	0.69
Shock Index	0.757 (0.646–0.847)	< 0.01
O2, Sat (%)	0.590 (0.452–0.719)	0.38
**Laboratory values**		
Troponin I (ng/ml)	0.509 (0.386–0.632)	0.93
Lactate levels (mg/dl)	0.693 (0.536–0.823)	0.03
P CO2 (mmHg)	0.526 (0.370–0.678)	0.13
P O2 (mmHg)	0.533 (0.377–0.685)	0.18
pH	0.651 (0.493–0.789)	0.11
Hemoglobin (g/dl)	0.527 (0.410–0.642)	0.75
**Echocardiography**		
RA (mm)	0.628 (0.478–0.761)	0.42
RV (mm)	0.587 (0.453–0.713)	0.49
RV-RA gradient (mmHg)	0.536 (0.388–0.680)	0.74
TAPSE (mm)	0.581 (0.440–0.713)	0.39

SBP – systolic blood pressure, DBP - diastolic blood pressure, HR - heart rate, P CO2 - partial pressure of carbon dioxide, P O2 - partial pressure of oxygen in the blood, RA – right atrium, RV – right ventricle, TAPSE - tricuspid annular plane systolic excursion.

Due to the more frequent association with cardiogenic shock (OR= 15, 95% CI 3.0-74.3, p=0.0006) and systolic blood pressure levels below 90 mmHg (OR=5.5, 95% CI 1.2-24.5, p=0.03), patients with syncope were more likely to require inotropic support (OR = 5.2, 95 % 1.1-23.7, p=0.03).

Patients with syncope had a higher PESI score (150.9 ± 51.1 versus 99.9 ± 30.1, p = 0.0009) and an increased risk of in-hospital mortality (OR= 4.5, 95% CI 1.1-17.6, p=0.03). The differences in mortality were not maintained medium-term (p=0.39).

Higher median lactate levels (21 (18 – 38) mg/dl versus 17 (12 – 32) mg/dl), a marker of tissue hypoperfusion, were observed in the syncope group, trending for statistical significance. A notable mention is that in our study population the troponin levels were not associated with the presence of syncope on admission (p=0.9).

On echocardiography, patients with syncope had shorter pulmonary artery acceleration time (65.2 ±14.72 versus 82.9 ± 23.09 msec, p=0.02) and lower median pulmonary artery peak velocity (0.52 (0.39; 0.57) versus 0.78 (0.50; 0.80) m/sec, p=0.04). No significant differences were found in the study group concerning right heart diameters or right ventricular dysfunction.

Regarding thrombus location, syncope was not associated with a central APE on chest CT examination (p=0.95).

In univariate analysis, syncope (OR 4.16, 95% CI 1.07 – 16.10, p = 0.036) as well as lactate levels (AUC 0.697, 95% CI 0.504 – 0.890, p = 0.05), right heart chambers’ dimensions (AUC 0.725, 95% CI 0.564 – 0.886, p = 0.037 for right atrial diameter and AUC 0.730, 95% CI 0.553 – 0.906, p= 0.034 for right ventricular diameter), presence of shock (OR 9.5, 95% CI 2.23 – 40.39, p = 0.002) and PESI score (AUC 0.748, 95% CI 0.613 – 0.884, p = 0.002) predicted in-hospital mortality.

In multivariate logistic regression, however, syncope lost the statistical significance as independent predictor of in-hospital mortality (HR 0.68, 95% CI 0.092 – 5.115, p = 0.714). Lactate levels (HR 1.054, 95% CI 1.001 – 1.109, p = 0.045) and PESI (HR 1.021, 95% CI 1.001 – 1.042, p = 0.04) were the only significant independent predictors. 

## Discussion

A growing body of evidence led to the assumption that the presentation of APE with syncope is associated with poorer outcome, regardless of the presence of hemodynamic instability [4,19]. But not all studies confirmed this association, especially in recent years [6,7,20].

The prevalence of syncope in our study was 14.3% in accordance with most percentages reported by other analyses. In contrast, the incidence of syncope was remarkably higher in patients with systolic arterial blood pressure below 90 mmHg (36.4%).

Additionally, in all our cases there was a robust temporal coherence between the syncope and the APE event (less than 24 hours), and so we acknowledged APE to be the trigger event of syncope. None of our patients had a previous syncopal episode; therefore, we did not consider it necessary to investigate additional underlying causes of syncope further.

Unlike Bülent et al. [23] we did not find an increased number of syncopal episodes in patients older than 60 (p=0.63) nor 65 years (p=0.65).

We found a strong association between syncope and cardiogenic shock and systolic blood pressure below 90 mmHg. Patients with syncope had a higher PESI score and a greater in-hospital mortality rate than the ones without this symptom at admission. This difference was not maintained on medium-term mortality rate (median surveillance 12 ± 1 month).

The mechanisms for syncope during APE are not completely understood [9,24]. The primary proposed mechanism is based on the presence of a large pulmonary artery embolus with greater than 50% occlusion of the pulmonary vascular tree accompanied by RVD and consecutively impaired left ventricular filling, leading to decreased cardiac output with arterial hypotension and declined cerebral blood flow resulting in syncope [19]. In our sample, the syncopal episode was not associated with a more central location of the thrombus load on chest CT examination, unlike other similar studies [22,23]. We, therefore, hypothesize that an equally important pathophysiological mechanism could be the partial resolution or displacement of the thrombus load, which might explain the incidence of syncope in our study population.

Another mechanism is based on the observation that APE could be associated with arrhythmias secondary to the right ventricle strain [25]. Although we found no electrocardiographic evidence of dysrhythmia on admission in our population, we cannot dismiss this hypothesis, since electrocardiographic tracings were not obtained immediately after the syncopal episode in all patients. Six patients presented with known atrial fibrillation, with a maximum heart rate of 130 bpm.

APE, partially occluding the pulmonary artery bed, could trigger a vasovagal reflex (Bezold-Jarisch reflex) resulting in a sudden decline of cardiac output, vasodilatation and neurocardiogenic syncope [2,25–27]. Due to the time elapsed between syncope and presentation to the hospital, although we could not accurately assess the implications of this mechanism in our patients, we consider it to be a possible cause of syncope.

Another important factor in the pathophysiology of syncope seems to be the hypoxemia secondary to ventilation or perfusion abnormalities [18]. However, the frequency of syncope among our patients did not differ with oxygen saturation.

A meta-analysis conducted by Becattini et al. in 2007 [28] on 1985 patients showed elevated concentrations of cardiac troponin I or T in approximately 50% of the patients with APE associated with prognostic implications. The elevated concentrations of troponins are a result of RV ischemia secondary to increased RV wall tension and neurohumoral activation. In our study, subjects with syncope did not have a significantly higher rate of troponin positivity.

The frequency of echocardiographic dysfunction defined by TAPSE below 16 mm was higher in the syncope group but without statistical significance (p= 0.56). In the study of Kasper et al., 35% of the enrolled high-risk APE patients or patients with RVD showed an APE-related syncope [3].

Syncope in APE patients has been proposed as an indicator of poor prognosis in the data reported by Thames et al. [4] or registries [17]. In ICOPER, the 3-month mortality rate of patients with syncope was 26.8%, compared to the overall mortality rate of 17% [17].

However, several recent studies [6,7,20] indicated that syncope is not related to mortality, but is related to hemodynamic instability, like in our study, offering no independent value over the clinical severity score.

Our results indicated that patients with syncope do not have a more centrally located thrombus, RVD, and troponin positivity. However, this condition was associated with the in-hospital mortality rate in our study population, probably as a marker of hemodynamic instability.

## Conclusion

Although syncope was significantly correlated with a higher in-hospital mortality rate and hemodynamic instability, the only independent predictors of mortality remain the PESI score and the presence of cardiogenic shock. In our sample, syncope did not reclassify the patient in a higher mortality group independently, but due to the association with hemodynamic instability, which remains, in our opinion, the primary indicator of poor prognosis. Evaluating the time between syncope and presentation as well as the number of these episodes can provide additional data in the future.

## Limitations

This study has several limitations. The design of our study was retrospective. The small number of patients in the syncope group was another factor limiting the interpretation of the results. We did not collect an extensive cardiopulmonary history to all of our patients.

## Conflict of Interest

The authors confirm that there are no conflicts of interest.
